# Rebamipide for management of methotrexate-induced oral ulcers: a three-arm randomized clinical trial

**DOI:** 10.1007/s00784-025-06159-x

**Published:** 2025-02-01

**Authors:** Amira Abdelaziz, Rehab Nabil Shamma, Hala Lotfy Fayed, Shereen Ali

**Affiliations:** 1 Department of Oral Medicine and Periodontology, Faculty of Dentistry, Cairo University, Giza, Egypt; 2 Department of Pharmaceutics and Industrial Pharmacy, Faculty of Pharmacy, Cairo University, Giza, Egypt; 3 Department of Rheumatology & Rehabilitation, Faculty of Medicine, Cairo University, Giza, Egypt; 4 Department of Oral Medicine and Periodontology, Faculty of Dentistry, Cairo University, 11 El-Saraya Street, Manial, 11553 Cairo Egypt

**Keywords:** Rebamipide, Nanoparticles, Corticosteroid, Methotrexate, Rheumatoid arthritis, Oral ulcers

## Abstract

**Objectives:**

This RCT aimed to evaluate the effect of topical Rebamipide (regular and nanoparticulated) in comparison to topical Clobetasol propionate in the management of methotrexate-induced oral ulcers in rheumatoid arthritis patients.

**Materials and methods:**

Patients were divided randomly into three parallel arms: 1% Rebamipide; 1% nanoparticulated Rebamipide, Clobetasol propionate. The outcome measures included WHO oral mucositis grading, pain (NRS), ulcer size, and healing time. The data was analyzed for any statistical significance.

**Results:**

Intragroup comparisons of mucositis grade improvement and pain reduction revealed significant differences in all the groups. All intergroup comparisons demonstrated non-significant difference, yet nanoparticulated Rebamipide was leading, and all group participants achieved complete healing earlier than the other groups.

**Conclusion:**

Rebamipide, regular and nanoparticulated forms, showed comparable results to potent Corticosteroid, Clobetasol propionate in management of the oral ulcers.

**Clinical relevance:**

Rebamipide is an efficient promising alternative modality for management of methotrexate-induced oral ulcers in rheumatoid arthritis patients.

**Supplementary information:**

The online version contains supplementary material available at 10.1007/s00784-025-06159-x.

## Introduction

Rheumatoid arthritis (RA) is a prevalent immune-mediated disorder [1]. The mainstays of RA treatment are early recognition and treatment with disease modifying anti-rheumatic drugs (DMARDs) [2]. Low-dose methotrexate (MTX) is a cornerstone DMARD that showed significant improvement of RA, yet painful oral ulcers are recurrent adverse effect [3].

RA is an interesting, good example to study the oral adverse effect, namely MTX-induced oral ulcers because oral ulcers have not been reported as a manifestation of the disease itself, so it is much easier to diagnose and monitor the drug induced oral ulcer [4].

MTX-induced oral ulcers can present in different forms including aphthous-like ulcers, deep irregular ulcers and diffuse mucositis [5]. Folic acid is the recommended antidote for MTX toxicity, a routine regimen of 5 mg folic acid can reduce MTX adverse effects including MTX-induced oral ulcers. Nevertheless, folic acid cannot always prevent the occurrence of oral ulcers or lessen its severity [6, 7].

Other approaches include cessation of MTX and changing or reducing the dose, but both carry other systemic risks. Treatment of oral ulcers was proposed using topical analgesics and topical corticosteroids. Yet, some cases are resistant to treatment, and some patients suffer from the side effects of corticosteroids [8, 9].

According to Ariyawardana et al. [10], many efficacious treatments for chemotherapy-induced oral mucositis were introduced for treatment and prevention of oral mucositis, among them Rebamipide (RB). Originally, RB was developed for the management of gastritis and gastric ulcers as a mucosal protection agent [11]. It helps in producing cytoprotective prostaglandins, reducing oxygen radicals, and increasing blood flow, thus enhancing the healing process [12].

Investigating the effect of RB was not restricted to the management of gastritis and gastric ulcers but extended to the management of stomatitis [12], drug-induced nephrotoxicity [13], and corneal protection [14]. Experimental studies proved that RB nanoforms are associated with improved penetration, stability, solubility, and mucoadhesive features, consequently improved healing [15, 16].

There is scarcity in the randomized clinical trials (RCTs) focusing on management of MTX-induced oral ulcers with only one clinical trial by Ahmed et al. [17]. The current trial was designed to assess the effect of topical RB (regular and nanoparticulated) compared to topical Clobetasol Propionate in management of MTX-induced oral ulcers in RA patients.

## Materials and methods

### Trial design

The current RCT is comparing three parallel arms with a 1:1:1 allocation ratio. The trial followed the principles of the Helsinki Declaration and was approved by the Research ethics committee of the Faculty of Dentistry, Cairo University (Code: 22920). The protocol was registered at ClinicalTrials.gov (NCT04649697).

### Participants

Participants were recruited from the Rheumatology clinics, Faculty of Medicine, Cairo University. Eligible participants were enrolled, according to the following criteria, in a consecutive order until the target sample was reached.

Inclusion criteria: RA patients treated with MTX and suffering from MTX-induced oral ulcers, age range (20–70 years old), patient who agreed to sign informed consent. Exclusion criteria: patients receiving treatment for the oral ulcers, patients suffering from malignancies, life-threatening systemic diseases or systemic diseases known to cause oral ulcers, patients with known or suspected history of hypersensitivity to interventions’ ingredients, pregnant or lactating females.

### Interventions

All interventions were in the form of mucoadhesive thermosensitive orabase hydrogel that was prepared using Chitosan and Beta-glycerol phosphate, as described by Haider et al. [18]. For RB loaded hydrogel, RB (Pharma Cure Pharmaceuticals, Egypt) was dissolved in cold chitosan hydrogel to achieve a concentration of 1% w/v following Chaitanya et al. [19]. For RB nanocrystals-loaded hydrogel, RB was prepared as nanocrystals using the sonocrystallization technique as described by Shamma & Latif [20]. Then, RB nanocrystals were dissolved in cold chitosan hydrogel to achieve a concentration of 1% w/v following Chaitanya et al. [19]. For Clobetasol propionate loaded hydrogel, Clobetasol (European Egyptian Pharmaceuticals, Egypt) was dissolved in cold chitosan hydrogel to achieve a concentration of 0.05% w/v following Ahmed et al. [17].

The three preparations had similar appearance, color, smell, weight, and taste with peach flavor to mask the bitter taste of RB. The prepared interventions were packed in sealed, identical, and opaque containers labelled A, B and C by a pharmacist who is not involved in the remaining steps of the trial.

Interventions were applied topically 4 times/day, after meals and before bedtime. Patients were instructed to refrain from eating or drinking for 1 h after applying the treatment. Nystatin 100,000 IU/cm3 in aqueous solution (Egyptian Int. Pharmaceuticals Ind Co. – Egypt) was prescribed as a mouth wash 4 times/day to avoid secondary fungal infection.

### Outcomes

The primary outcome was clinical improvement of oral ulcers assessed using WHO oral mucositis grading. Grade of mucositis were given score from 0 to 4: Grade 0 = No oral mucositis; Grade 1 = Erythema and soreness; Grade 2 = Ulcers, able to eat solids; Grade 3 = Ulcers, requires liquid diet (due to mucositis); Grade 4 = Ulcers, alimentation not possible (due to mucositis) [21, 22].

The secondary outcomes included pain assessed using numerical rating scale (NRS) consisting of a 10-cm horizontal line between extremities with (0) indicating no pain and (10) for unbearable pain [23], ulcer size determined by measuring the distance between two opposite edges of the ulcer border, using a periodontal probe in millimeters and healing time was recorded in days. All outcomes were measured at baseline and weekly by the outcome assessor, additionally NRS was assessed daily during the first week by the patients. Patients received a demonstration about NRS and how to express their pain through it. Patients were instructed to fill in the NRS follow-up card daily and they received a daily reminder to fill the card.

### Sample size

Sample size was determined based on WHO mucositis scale improvement of Ahmed et al. [17]. Using power 80% and 5% Alpha significance level, 11 participants are required in each group. This number was increased to 13 to compensate for follow-up losses. Sample size calculation was conducted using PS: Power and Sample Size Calculation Software Version 3.1.2 (Vanderbilt University, Nashville, TN, USA). The sample size calculation was revised and approved by the Medical Biostatistics Unit of the Faculty of Dentistry, Cairo University.

### Randomization and blinding

Simple randomization was generated using http://www.random.org by the statistician, who then concealed the sequence in numbered, opaque, sealed envelopes. After finishing baseline assessments, the envelopes were opened to allocate the intervention. The participants, care provider and the outcome assessor were blinded to the intervention. The three interventions were prepared to have similar characteristics, as detailed in the intervention section, and were packed in identical opaque containers labelled A, B and C by a pharmacist who is not involved in the remaining steps of the trial.

### Statistical methods

Categorical data were presented as frequency and percentage values and were analyzed using Fisher’s exact test. Numerical data were presented as mean, standard deviation (SD), median, and interquartile range (IQR) values. They were analyzed for normality using Shapiro-Wilk’s test. Age data were analyzed using a one-way ANOVA test. Other data were analyzed using Kruskal-Wallis’s test followed by Dunn’s post hoc test for intergroup comparisons and Friedman’s test followed by Nemenyi’s post hoc test for intragroup comparisons. P-values were adjusted for multiple comparisons using False Discovery Rate (FDR) method. The significance level was set at *p* < 0.05. Statistical analysis was performed with R statistical analysis software version 4.3.2 for Windows.

## Results

**Fig. 1:**
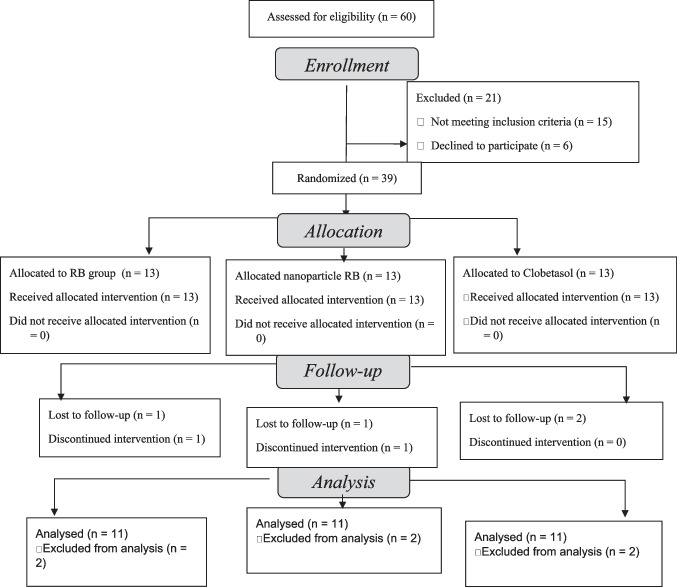
Consort flow diagram

 Tables 1 and S1 demonstrate baseline demographic and clinical characteristics. The total sample size comprised 39 participants. Two participants discontinued the treatment, and four participants dropped off during the follow-ups (Fig. 1).

**Table 1:** Baseline demographic and clinical characteristics

		RB	Nanoparticulated RB	Clobetasol	*p*-value
**Gender #**	**Male**	2 (18.18%)	1 (9.09%)	1 (9.09%)	**1**
	**Female**	9 (81.82%)	10 (90.91%)	10 (90.91%)	
**Age (years)**	**Mean ± SD**	49.45 ± 17.18	46.27 ± 12.59	48.45 ± 13.40	**0.982**
	**Median (IQR)**	42.00(26.50)	50.00(10.50)	45.00 (15.50)	
**MTX dose (ml/week)**	**Mean ± SD**	18.64 ± 4.52	17.50 ± 3.35	17.50 ± 4.61	**0.829**
	**Median (IQR)**	17.50 (7.50)	17.50 (5.00)	17.50 (6.25)	
**MTX duration (years)**	**Mean ± SD**	5.64 ± 6.30	9.18 ± 10.80	3.64 ± 4.67	**0.091**
	**Median (IQR)**	3.00 (5.00)	3.00 (8.00)	1.00 (2.50)	
**Ulcer size (mm)**	**Mean ± SD**	83.23 ± 196.97	42.27 ± 86.97	157.95 ± 276.68	**0.443**
	**Median (IQR)**	20.00 (23.75)	12.00 (15.50)	14.00 (175.00)	
**Mucositis Score**	**Mean ± SD**	2.82 ± 0.60	2.36 ± 0.50	2.73 ± 0.47	**0.123**
	**Median (IQR)**	3.00 (0.50)	2.00 (1.00)	3.00 (0.50)	
**Pain score**	**Mean ± SD**	6.55 ± 1.86	6.00 ± 2.10	7.55 ± 1.92	**0.206**
	**Median (IQR)**	6.00 (2.50)	6.00 (2.00)	7.00 (2.50)	

#: Count and percentage

 Concerning WHO mucositis scores, all intragroup comparisons showed a significant reduction by time. The three arms demonstrated a significant reduction between the baseline and the first week. For RB groups, the reduction continued without statistical significance through the remaining weeks, all the patients reached complete healing by the fourth week. For Clobetasol group, a significant reduction was detected between the first and the third weeks (Tables 2 & S1).

**Table 2:** Intragroup comparisons of WHO mucositis scores and ulcer size (mm^2^)

		WHO mucositis scores			Ulcer size (mm^2^)		
		RB	Nanoparticulated RB	Clobetasol	RB	Nanoparticulated RB	Clobetasol
**Baseline**	**Mean ± SD**	2.82 ± 0.60^a^	2.36 ± 0.50^a^	2.73 ± 0.47^a^	83.23 ± 196.97^a^	42.27 ± 86.97^a^	157.95 ± 276.68^a^
	**Median (IQR)**	3.00 (0.50)^a^	2.00 (1.00)^a^	3.00 (0.50)^a^	20.00 (23.75)^a^	12.00 (15.50)^a^	14.00 (175.00)^a^
**Week 1**	**Mean ± SD**	0.55 ± 1.04^b^	0.36 ± 0.67^b^	0.82 ± 1.25^b^	16.55 ± 54.22^a^	9.82 ± 31.58^a^	33.45 ± 74.21^ab^
	**Median (IQR)**	0.00 (0.50)^b^	0.00 (0.50)^b^	0.00 (1.50)^b^	0.00 (0.00)^a^	0.00 (0.00)^a^	0.00 (21.00)^ab^
**Week 2**	**Mean ± SD**	0.27 ± 0.90^b^	0.00 ± 0.00^b^	0.55 ± 0.93^bc^	0.91 ± 3.02^a^	0.00 ± 0.00^a^	0.82 ± 1.40^b^
	**Median (IQR)**	0.00 (0.00)^b^	0.00 (0.00)^b^	0.00 (1.00)^bc^	0.00 (0.00)^a^	0.00 (0.00)^a^	0.00 (1.50)^b^
**Week 3**	**Mean ± SD**	0.09 ± 0.30^b^	0.00 ± 0.00^b^	0.18 ± 0.60^bc^	0.00 ± 0.00^a^	0.00 ± 0.00^a^	0.27 ± 0.90^b^
	**Median (IQR)**	0.00 (0.00)^b^	0.00 (0.00)^b^	0.00 (0.00)^bc^	0.00 (0.00)^a^	0.00 (0.00)^a^	0.00 (0.00)^b^
**Week 4**	**Mean ± SD**	0.00 ± 0.00^b^	0.00 ± 0.00^b^	0.00 ± 0.00^c^	0.00 ± 0.00^a^	0.00 ± 0.00^a^	0.00 ± 0.00^b^
	**Median (IQR)**	0.00 (0.00)^b^	0.00 (0.00)^b^	0.00 (0.00)^c^	0.00 (0.00)^a^	0.00 (0.00)^a^	0.00 (0.00)^b^
*p* -value		< 0.001*	< 0.001*	< 0.001*	**0.117**	**0.053**	0.014*

*: Significant at *P* ≤ 0.05; Lowercase letters denote significance within columns

 Intergroup comparisons revealed no significant difference for all time points. At baseline, RB scored the highest value followed by Clobetasol then nanoparticulated RB. At the first week, Clobetasol recorded the highest score followed by RB, then nanoparticulated RB. At the second and the third weeks, Clobetasol scores remained higher than RB. All nanoparticulated RB participants achieved complete healing early by the second week. Then, patients of RB and Clobetasol reached complete healing by the fourth week (Table 3 & S2).

**Table 3:** Intergroup comparisons of WHO mucositis scores and ulcer size (mm^2^)

		WHO mucositis scores				Ulcer size (mm^2^)			
		RB	Nanoparticulated RB	Clobetasol	*p*-value	RB	Nanoparticulated RB	Clobetasol	*p*-value
**Baseline**	**Mean ± SD**	2.82 ± 0.60^A^	2.36 ± 0.50^A^	2.73 ± 0.47^A^	**0.123**	83.23 ± 196.97^A^	42.27 ± 86.97^A^	157.95 ± 276.68^A^	**0.443**
	**Median (IQR)**	3.00 (0.50)^A^	2.00 (1.00)^A^	3.00 (0.50)^A^		20.00 (23.75)^A^	12.00 (15.50)^A^	14.00 (175.00)^A^	
**Week 1**	**Mean ± SD**	0.55 ± 1.04^A^	0.36 ± 0.67^A^	0.82 ± 1.25^A^	**0.755**	16.55 ± 54.22^A^	9.82 ± 31.58^A^	33.45 ± 74.21^A^	**0.786**
	**Median (IQR)**	0.00 (0.50)^A^	0.00 (0.50)^A^	0.00 (1.50)^A^		0.00 (0.00)^A^	0.00 (0.00)^A^	0.00 (21.00)^A^	
**Week 2**	**Mean ± SD**	0.27 ± 0.90^A^	0.00 ± 0.00^A^	0.55 ± 0.93^A^	**0.168**	0.91 ± 3.02^A^	0.00 ± 0.00^A^	0.82 ± 1.40^A^	**0.168**
	**Median (IQR)**	0.00 (0.00)^A^	0.00 (0.00)^A^	0.00 (1.00)^A^		0.00 (0.00)^A^	0.00 (0.00)^A^	0.00 (1.50)^A^	
**Week 3**	**Mean ± SD**	0.09 ± 0.30^A^	0.00 ± 0.00^A^	0.18 ± 0.60^A^	**0.596**	0.00 ± 0.00^A^	0.00 ± 0.00^A^	0.27 ± 0.90^A^	**0.368**
	**Median (IQR)**	0.00 (0.00)^A^	0.00 (0.00)^A^	0.00 (0.00)^A^		0.00 (0.00)	0.00 (0.00)	0.00 (0.00)	
**Week 4**	**Mean ± SD**	0.00 ± 0.00	0.00 ± 0.00	0.00 ± 0.00	**NA**	0.00 ± 0.00	0.00 ± 0.00	0.00 ± 0.00	**NA**
	**Median (IQR)**	0.00 (0.00)	0.00 (0.00)	0.00 (0.00)		0.00 (0.00)	0.00 (0.00)	0.00 (0.00)	

Uppercase letters denote significance within rows

Regarding ulcer size, intragroup comparison showed a significant decrease in size over time in all groups. For RB groups, there was no statistical significance between the different visits. For Clobetasol group, a significant reduction was spotted between the baseline and the second week (Table 2).

Intergroup comparison revealed no significant difference at any time point. At the baseline and the first week, Clobetasol scored the highest value, followed by RB, then nanoparticulated RB. At the second week, RB and Clobetasol were comparable, while all nanoparticulated RB cases attained complete resolution. RB participants followed them by the third week and Clobetasol participants reached complete healing by the fourth week (Table 3).

 The shortest healing time was recorded by nanoparticulated RB, followed by RB, then Clobetasol with no statistical difference between the three arms (Table 4).

**Table 4:** Intergroup comparison of healing time (days)

	RB	Nanoparticulated RB	Clobetasol	*p*-value
**Mean ± SD**	5.82 ± 5.25^A^	5.55 ± 4.11^A^	8.73 ± 7.25^A^	**0.238**
**Median (IQR)**	4.00 (1.50)^A^	4.00 (2.00)^A^	5.00 (7.00)^A^	

*: Significant at *P* ≤ 0.05; Uppercase letters denote significance within row

 As regards pain scores, all intragroup comparisons demonstrated a significant reduction over time. In all the groups, the first day scores were significantly higher than other intervals except for the second day. Plus, the second day’s scores were significantly higher than those reported starting from the fourth day, the third day’s scores were significantly higher than those reported starting from the fifth day, and the fourth day’s scores were significantly higher than those reported starting from the sixth day. Finally, the fifth day’s scores were significantly higher than those reported starting from the second week. Only in Clobetasol group, weekly pain scores showed significant reduction (Table 5).

**Table 5:** Intragroup comparisons of pain score

		RB	nanoparticulated RB	Clobetasol
**Baseline/Day 1**	**Mean ± SD**	6.55 ± 1.86^a^	6.00 ± 2.10^a^	7.55 ± 1.92^a^
	**Median (IQR)**	6.00 (2.50)^a^	6.00 (2.00)^a^	7.00 (2.50)^a^
**Day 2**	**Mean ± SD**	5.55 ± 2.38^ab^	4.73 ± 2.10^ab^	6.64 ± 1.96^ab^
	**Median (IQR)**	6.00 (2.00)^ab^	5.00 (3.50)^ab^	6.00 (3.00)^ab^
**Day 3**	**Mean ± SD**	4.18 ± 2.27^bc^	3.45 ± 2.07^bc^	5.55 ± 2.11^bc^
	**Median (IQR)**	4.00 (2.00)^bc^	3.00 (3.00)^bc^	5.00 (2.50)^bc^
**Day 4**	**Mean ± SD**	3.09 ± 2.47^cd^	2.36 ± 2.11^cd^	4.09 ± 2.55^cd^
	**Median (IQR)**	3.00 (2.50)^cd^	1.00 (3.00)^cd^	4.00 (3.50)^cd^
**Day 5**	**Mean ± SD**	2.18 ± 2.56^de^	1.55 ± 1.92^de^	3.18 ± 2.60^de^
	**Median (IQR)**	1.00 (1.50)^de^	0.00 (3.00)^de^	3.00 (3.50)^de^
**Day 6**	**Mean ± SD**	1.27 ± 2.37^ef^	0.91 ± 1.38^ef^	2.09 ± 2.51^ef^
	**Median (IQR)**	0.00 (1.50)^ef^	0.00 (1.00)^ef^	1.00 (3.00)^ef^
**Day 7/ Week 1**	**Mean ± SD**	1.00 ± 2.41^ef^	0.36 ± 0.67^ef^	1.64 ± 2.54^efg^
	**Median (IQR)**	0.00 (0.50)^ef^	0.00 (0.50)^ef^	0.00 (3.00)^efg^
**Week 2**	**Mean ± SD**	0.45 ± 1.51^f^	0.00 ± 0.00^f^	0.55 ± 0.93^fgh^
	**Median (IQR)**	0.00 (0.00)^f^	0.00 (0.00)^f^	0.00 (1.00)^fgh^
**Week 3**	**Mean ± SD**	0.09 ± 0.30^f^	0.00 ± 0.00^f^	0.18 ± 0.60^gh^
	**Median (IQR)**	0.00 (0.00)^f^	0.00 (0.00)^f^	0.00 (0.00)^gh^
**Week 4**	**Mean ± SD**	0.00 ± 0.00^f^	0.00 ± 0.00^f^	0.00 ± 0.00^h^
	**Median (IQR)**	0.00 (0.00)^f^	0.00 (0.00)^f^	0.00 (0.00)^h^
***p*-value**		< 0.001*	< 0.001*	< 0.001*

*: Significant at *P* ≤ 0.05; Lowercase letters denote significance within columns

 Intergroup comparison revealed no significant difference between pain scores at any time point. Clobetasol participants reported the highest scores, followed by RB, then nanoparticulated RB at all visits. All nanoparticulated RB patients reported zero pain score early by the second week, followed by RB and Clobetasol patients by the fourth week (Table 6).

**Table 6:** Intergroup comparisons of pain score

		RB	nanoparticulated RB	Clobetasol	*p*-value
**Baseline/Day 1**	**Mean ± SD**	6.55 ± 1.86^A^	6.00 ± 2.10^A^	7.55 ± 1.92^A^	**0.206**
	**Median (IQR)**	6.00 (2.50)^A^	6.00 (2.00)^A^	7.00 (2.50)^A^	
**Day 2**	**Mean ± SD**	5.55 ± 2.38^A^	4.73 ± 2.10^A^	6.64 ± 1.96^A^	**0.190**
	**Median (IQR)**	6.00 (2.00)^A^	5.00 (3.50)^A^	6.00 (3.00)^A^	
**Day 3**	**Mean ± SD**	4.18 ± 2.27^A^	3.45 ± 2.07^A^	5.55 ± 2.11^A^	**0.110**
	**Median (IQR)**	4.00 (2.00)^A^	3.00 (3.00)^A^	5.00 (2.50)^A^	
**Day 4**	**Mean ± SD**	3.09 ± 2.47^A^	2.36 ± 2.11^A^	4.09 ± 2.55^A^	**0.246**
	**Median (IQR)**	3.00 (2.50)^A^	1.00 (3.00)^A^	4.00 (3.50)^A^	
**Day 5**	**Mean ± SD**	2.18 ± 2.56^A^	1.55 ± 1.92^A^	3.18 ± 2.60^A^	**0.220**
	**Median (IQR)**	1.00 (1.50)^A^	0.00 (3.00)^A^	3.00 (3.50)^A^	
**Day 6**	**Mean ± SD**	1.27 ± 2.37^A^	0.91 ± 1.38^A^	2.09 ± 2.51^A^	**0.455**
	**Median (IQR)**	0.00 (1.50)^A^	0.00 (1.00)^A^	1.00 (3.00)^A^	
**Day 7/ Week 1**	**Mean ± SD**	1.00 ± 2.41^A^	0.36 ± 0.67^A^	1.64 ± 2.54^A^	**0.683**
	**Median (IQR)**	0.00 (0.50)^A^	0.00 (0.50)^A^	0.00 (3.00)^A^	
**Week 2**	**Mean ± SD**	0.45 ± 1.51^A^	0.00 ± 0.00^A^	0.55 ± 0.93^A^	**0.168**
	**Median (IQR)**	0.00 (0.00)^A^	0.00 (0.00)^A^	0.00 (1.00)^A^	
**Week 3**	**Mean ± SD**	0.09 ± 0.30^A^	0.00 ± 0.00^A^	0.18 ± 0.60^A^	**0.596n**
	**Median (IQR)**	0.00 (0.00)^A^	0.00 (0.00)^A^	0.00 (0.00)^A^	
**Week 4**	**Mean ± SD**	0.00 ± 0.00^A^	0.00 ± 0.00^A^	0.00 ± 0.00^A^	**NA**
	**Median (IQR)**	0.00 (0.00)^A^	0.00 (0.00)^A^	0.00 (0.00)^A^	

*: Significant at *P* ≤ 0.05; Uppercase letters denote significance within rows

## Discussion

Drug-induced oral ulcers are painful, troublesome and may worsen patient’s condition progressively, prompting doses adjustments or possibly medication change [24]. Management of drug-induced oral ulcers is a deficient research area, especially in RA patients who are exposed to MTX toxicity. Management of MTX-induced oral ulcers is complicated and challenging as ulcers do not respond well to traditional treatments like corticosteroids. Altering the dosage, quitting therapy, or switching medications may be the choice, with their potential drawbacks [8, 9].

RB, a quinolinone amino acid analog, is among assessed therapies in the prevention and treatment of chemotherapy-induced oral mucositis [10]. Furthermore, RB was assessed in several oral purposes with positive outcomes including Sjögren syndrome [25, 26], recurrent aphthous ulcer (RAU) [27, 28], and oral aphthous like ulcer of Behçet’s disease [29].

Studies on artificial oral mucosa and animal models using the nanoparticulated RB showed impressive results in healing of oral wounds [15, 16]. The nanoparticulated RB, loaded on poly (DL-lactide-co-glycolide) and coated with chitosan, proved effective in healing of chemotherapy-induced oral mucositis in mouse models [30].

Accordingly, this study was designed to provide a safe alternative for management of MTX-induced oral ulcers. As far as we know, the current RCT is the first to study the effect of RB on low dose MTX-induced oral ulcers. Besides, it is the first study to investigate the effect of nanoparticulated RB on oral ulcers in human subjects.

All the participants were receiving low dose MTX with folic acid supplementation. Yet, they experienced oral ulcers. This was in line with a Cochrane review by Shea et al. [31], the review documented that there was no significant effect of folic acid intake in reducing the incidence of oral ulcers.

In the present trial, the participants were recruited from one public hospital to ensure the same socioeconomic level, thus avoiding potential confounders. The participants were enrolled consecutively to avoid selection bias. The aim and the procedures of the study were explained to the participants to avoid non-respondent bias.

Concerning the results of WHO mucositis grading, there was a significant reduction from baseline to the end of the trial within all the groups. However, there was no significant difference between the effect of RB and Clobetasol. Still, these results demonstrate the positive comparable effect of RB in front of Clobetasol, one of the most potent Corticosteroids.

The beneficial effect of RB in management of drug-induced oral mucositis was addressed by Akagi et al. [32]. They documented that RB mouthwash reduced sever chemotherapy-induced oral mucositis (grade 3 and 4). The systematic review included three RCTs. Yokota et al. [33] stated that the effect of 4% RB mouthwash was better than 2% RB and placebo in reducing oral mucositis, yet with no statistical significance. On the other hand, Yasuda et al. [11], and Chaitanya et al. [19] agreed that 1% RB gargle significantly reduced the incidence of severe oral mucositis compared to placebo. The three RCTs prescribed RB six times per day. The current RCT investigated the lowest concentration (1%) of RB, as a mucoadhesive gel, four times per day for better compliance.

Viewing the current results of nanoparticulated RB, all the participants reached complete healing after two weeks compared to four weeks in the other groups. Moreover, the healing time was the shortest without significant difference. These results should be taken with cautiousness because mean and median scores of nanoparticulated RB were the lowest at baseline, with no statistical significance and many participants were suffering from grade 2 oral mucositis.

The positive effect of RB nanoparticles was documented in animal studies. Nakashima et al. [34] detected significant effect of 2% RB submicronized crystals suspension with moderate viscosity against its control on improvement of cauterization-induced oral ulcer. Nagai et al. [35] studied oral RB for treatment of indomethacin-induced injuries in gastrointestinal mucosa of rats. They found that the healing effect of RB nanoparticles was significantly higher than traditional RB and that there was a significant increase in drug concentration of RB nanocrystals by time compared to traditional RB.

Back to the current RCT, RB (regular and nanoparticulated) showed a non-significant decrease in ulcer size by time. In contrast, Clobetasol showed a statistically significant decrease in ulcer size. This can be attributed to the small average ulcer size in RB groups compared to Clobetasol group, despite the non-significant difference in ulcer size between the groups at baseline.

Earlier studies investigated the effect of RB on the ulcer size of RAU. Parvathi Devi et al. [27] stated that RB and Levamisole were effective in reducing ulcer size with no significant difference between them. Hasan et al. [28] found that RB tablets combined with Amlexanox paste lead to a significant decrease in ulcer size compared to topical Dologel CT. Moreover, two animal studies noted that 1%, 2% and 4% RB submicronized crystals suspension produced a significant decrease in ulcer size in comparison to the control in mouse models [34, 36].

In the current RCT, there was a trend towards shortening of healing time by RB, with nanoparticulated RB showing the shortest healing time followed by regular RB, then Clobetasol without significant difference. Takeuchi et al. [37] found a significant difference between chitosan-coated RB nanoparticles suspension compared to regular RB and RB nanoparticles suspensions without chitosan in decreasing healing time. They attributed this difference to the chitosan that increased the mucoadhesive properties which subsequently improved the therapeutic effect and shortened the healing time. This could clarify the non-significant difference between our study groups as all interventions included chitosan, thus have the same retention capacity.

The current results revealed a significant pain reduction in each of the three groups. However, the pain reduction was not significantly different in-between the groups. Parvathi Devi et al. [27] showed that RB and Levamisole reduced pain of RAU with no significant difference. Other studies documented significant pain reduction associated with RB compared to placebo in management of aphthous like ulcers of Behcet’s disease [29], and chemoradiotherapy-induced oral mucositis [19].

In the current study, no harmful adverse effects were reported. One patient in Clobetasol group and another patient in RB group reported a fungal infection that resolved with antifungal treatment within a week.

In brief, even though the intergroup statistical comparisons revealed non-significant differences, all the interventions showed effectiveness in management of oral ulcers. RB in its two forms, the regular and the nanoparticulated, demonstrated a slight clinical superiority compared to Clobetasol. Interestingly, this superior effect was demonstrated by a low concentration of RB (1%).

The interventions were applied 4 times daily, the patient received regular reminder by phone every other day to avoid any non-compliance. Another limitation was the sample size; we recommend larger sample size to allow subgroup analysis between different forms of MTX-induced oral ulcers. The strength of the current trial is being the first to study the effect of RB on the MTX-induced oral ulcers in RA population and the first study to assess nanoparticulated RB on human subjects.

## Conclusion

RB seems an efficient promising alternative modality for management of MTX- induced oral ulcers in RA patients. RB, regular and nanoparticulated forms, showed promising and comparable results to potent Corticosteroid, Clobetasol propionate. The authors recommend conducting studies with larger sample sizes to confirm the efficiency of RB. In addition, the authors recommend assessing different viscosities, concentrations, and formulations of RB.

## Supplementary Information

Below is the link to the electronic supplementary material.

ESM 1 (DOCX 17.4 KB)

ESM 2 (DOCX 17.6 KB)

## Data Availability

No datasets were generated or analysed during the current study.
