# Supplementary material for: Rebamipide for management of methotrexate-induced oral ulcers: a three-arm randomized clinical trial
Source: Clin Oral Investig. 2025 Feb 1;29(2):106. doi: 10.1007/s00784-025-06159-x (PMC11787234; doi:10.1007/s00784-025-06159-x)
Supplement: Supplementary file 1 — (DOCX 17.4 KB) [file 784_2025_6159_MOESM1_ESM.docx]

**Table (S1):** Detailed data of MTX dose and duration among the participants

| **Patient number** | **MTX dose (mg/week)** | | | **MTX duration (years)** | | |
| --- | --- | --- | --- | --- | --- | --- |
|  | **RB** | **nanoparticulated RB** | **Clobetasol** | **RB** | **nanoparticulated RB** | **Clobetasol** |
| 1 | 15 | 20 | 20 | 2 | 2 | 2 |
| 2 | 25 | 20 | 17.5 | 7 | 2 | 1 |
| 3 | 25 | 15 | 25 | 15 | 10 | 5 |
| 4 | 20 | 15 | 12.5 | 1 | 10 | 1 |
| 5 | 17.5 | 22.5 | 15 | 20 | 3 | 1 |
| 6 | 17.5 | 20 | 17.5 | 4 | 2 | 10 |
| 7 | 12.5 | 17.5 | 12.5 | 3 | 2 | 2 |
| 8 | 17.5 | 20 | 15 | 6 | 2 | 1 |
| 9 | 25 | 17.5 | 20 | 1 | 8 | 15 |
| 10 | 15 | 12.5 | 25 | 1 | 30 | 1 |
| 11 | 15 | 12.5 | 12.5 | 2 | 30 | 1 |
| **Mean±SD** | 18.64±4.52 | 17.50±3.35 | 17.50±4.61 | 5.64±6.30 | 9.18±10.80 | 3.64±4.67 |
| **Median (IQR)** | 17.50 (7.50) | 17.50 (5.00) | 17.50 (6.25) | 3.00 (5.00) | 3.00 (8.00) | 1.00 (2.50) |

MTX: Methotrexate

All participants were using folic acid as a part of the therapeutic regimen prescribed by the Rheumatology department.
