# Supplementary material for: Rebamipide for management of methotrexate-induced oral ulcers: a three-arm randomized clinical trial
Source: Clin Oral Investig. 2025 Feb 1;29(2):106. doi: 10.1007/s00784-025-06159-x (PMC11787234; doi:10.1007/s00784-025-06159-x)
Supplement: Supplementary file 2 — (DOCX 17.6 KB) [file 784_2025_6159_MOESM2_ESM.docx]

**Table (S2):** Intragroup and intergroup comparisons of WHO mucositis grades

| **Visit** | **Score** | **RB** | **nanoparticulated RB** | **Clobetasol** | **p-value** |
| --- | --- | --- | --- | --- | --- |
| **Baseline** | **0** | 0 (0.00%)^Aa^ | 0 (0.00%)^Aa^ | 0 (0.00%)^Aa^ | **0.123** |
|  | **1** | 0 (0.00%) | 0 (0.00%) | 0 (0.00%) |  |
|  | **2** | 3 (27.27%) | 7 (63.64%) | 3 (27.27%) |  |
|  | **3** | 7 (63.64%) | 4 (36.36%) | 8 (72.73%) |  |
|  | **4** | 1 (9.09%) | 0 (0.00%) | 0 (0.00%) |  |
| **Week 1** | **0** | 8 (72.73%)^Ab^ | 8 (72.73%)^Ab^ | 7 (63.64%)^Ab^ | **0.755** |
|  | **1** | 1 (9.09%) | 2 (18.18%) | 1 (9.09%) |  |
|  | **2** | 1 (9.09%) | 1 (9.09%) | 1 (9.09%) |  |
|  | **3** | 1 (9.09%) | 0 (0.00%) | 2 (18.18%) |  |
|  | **4** | 0 (0.00%) | 0 (0.00%) | 0 (0.00%) |  |
| **Week 2** | **0** | 10 (90.91%)^Ab^ | 11 (100.00%)^Ab^ | 8 (72.73%)^Abc^ | **0.168** |
|  | **1** | 0 (0.00%) | 0 (0.00%) | 0 (0.00%) |  |
|  | **2** | 0 (0.00%) | 0 (0.00%) | 3 (27.27%) |  |
|  | **3** | 1 (9.09%) | 0 (0.00%) | 0 (0.00%) |  |
|  | **4** | 0 (0.00%) | 0 (0.00%) | 0 (0.00%) |  |
| **Week 3** | **0** | 10 (90.91%)^Ab^ | 11 (100.00%)^Ab^ | 10 (90.91%)^Abc^ | **0.596** |
|  | **1** | 1 (9.09%) | 0 (0.00%) | 0 (0.00%) |  |
|  | **2** | 0 (0.00%) | 0 (0.00%) | 1 (9.09%) |  |
|  | **3** | 0 (0.00%) | 0 (0.00%) | 0 (0.00%) |  |
|  | **4** | 0 (0.00%) | 0 (0.00%) | 0 (0.00%) |  |
| **Week 4** | **0** | 11 (100.00%)^Ab^ | 11 (100.00%)^Ab^ | 11 (100.00%)^Ac^ | **NA** |
|  | **1** | 0 (0.00%) | 0 (0.00%) | 0 (0.00%) |  |
|  | **2** | 0 (0.00%) | 0 (0.00%) | 0 (0.00%) |  |
|  | **3** | 0 (0.00%) | 0 (0.00%) | 0 (0.00%) |  |
|  | **4** | 0 (0.00%) | 0 (0.00%) | 0 (0.00%) |  |
| **p-value** | | **<0.001*** | **<0.001*** | **<0.001*** |  |

#: Count and percentage; *: Significant at P ≤ 0.05

Uppercase letters denote significance within rows; Lowercase letters denote significance within columns
